# Macrogenomics Reveals Effects on Marine Microbial Communities during *Oplegnathus punctatus* Enclosure Farming

**DOI:** 10.3390/biology13080618

**Published:** 2024-08-15

**Authors:** Lijun Wang, Xiaofei Lu, Zhikai Xing, Xindong Teng, Shuang Wang, Tianyi Liu, Li Zheng, Xumin Wang, Jiangyong Qu

**Affiliations:** 1 College of Life Science, Yantai University, Yantai 264005, China; wanglijun@ytu.edu.cn (L.W.); luxiaofei18@outlook.com (X.L.); xingzhk@ytu.edu.cn (Z.X.); wangshuang0456@126.com (S.W.); liutianyi0719@163.com (T.L.); 2 Qingdao International Travel Healthcare Center, Qingdao 266071, China; tengxindeng@163.com; 3 First Institute of Oceanography, Ministry of Natural Resources, Qingdao 266061, China; zhengli@fio.org.cn; 4 Laboratory of Marine Ecology and Environmental Science, Qingdao National Laboratory for Marine Science and Technology, Qingdao 266061, China

**Keywords:** microbial community, aquaculture, antibiotic resistance gene, metagenomes, *Oplegnathus punctatus*

## Abstract

**Simple Summary:**

The water environment in Laizhou Bay has experienced mild eutrophication in recent years, impacting the interactions between environmental factors, microorganisms, and aquatic species. The metagenomic analysis of 142 samples from various water layers revealed that *Oplegnathus punctatus* mariculture significantly increases microbial abundance and complexity in the culture area. These alterations were more noticeable in sediments compared to seawater. Cyanobacteria and Candidatus Micrarchaeota in seawater, and Actinobacteria and Thaumarchaeota in sediments show notably higher abundances in the mariculture area, indicating shifts in microbial community structure due to aquaculture practices. Antibiotic resistance genes (ARGs) in sediments are highly sensitive to environmental variables, particularly oxygen solubility and salinity. This indicates that *Oplegnathus punctatus* mariculture can impact the occurrence and spread of antibiotic resistance in marine ecosystems. The study underscores the intricate and dynamic relationships between microorganisms, environmental conditions, and ARGs, highlighting the need for region-specific strategies in managing marine resources sustainably.

**Abstract:**

(1) Background: Laizhou Bay is an important aquaculture area in the north of China. *Oplegnathus punctatus* is one of the species with high economic benefits. In recent years, the water environment of Laizhou Bay has reached a mild eutrophication level, while microorganisms are an important group between the environment and species. In this study, we evaluated alterations in environmental elements, microbial populations, and antibiotic resistance genes (ARGs) along with their interconnections during *Oplegnathus punctatus* net culture. (2) Methods: A total of 142 samples from various water layers were gathered for metagenome assembly analysis. Mariculture increases the abundance of microorganisms in this culture area and makes the microbial community structure more complex. The change had more significant effects on sediment than on seawater. (3) Results: Certain populations of cyanobacteria and Candidatus Micrarchaecta in seawater, and Actinobacteria and Thaumarchaeota in sediments showed high abundance in the mariculture area. Antibiotic resistance genes in sediments were more sensitive to various environmental factors, especially oxygen solubility and salinity. (4) Conclusions: These findings highlight the complex and dynamic nature of microorganism–environment–ARG interactions, characterized by regional specificity and providing insights for a more rational use of marine resources.

## 1. Introduction

The Food and Agriculture Organization of the United Nations (FAO) states that China has surpassed the rest of the world in terms of aquacultural production since 1991 and currently holds a 60% share of global production [1]. The four primary provinces for aquaculture production, Guangdong, Shandong, Fujian, and Jiangsu, are situated in coastal regions and are predominantly focused on mariculture. Mariculture mainly includes land-based and nearshore sea aquaculture, with water depths in the nearshore sea areas typically not surpassing 15 m. The species is farmed in nets within the water [2]. However, the availability of suitable offshore areas for aquaculture is diminishing due to the impact of the market economy. The highest aquaculture densities reached 17.7 tons/ha [2,3]. High-density aquaculture offers substantial potential for the rapid proliferation of pathogenic organisms [4]. This has led to increased environmental deterioration and frequent disease epidemics, posing significant challenges to the advancement of the fishery industry and conflicting with the goal of sustainable development.

Artificial aquaculture practices exert considerable pressure on cultivated fish, compromising their natural immune system’s ability to combat diverse bacterial and viral infections [5]. Furthermore, notable changes occurred in the diversity of environmental microbial communities and their biochemical parameters [6,7]. Under suboptimal conditions, specific groups of microorganisms may enter a dormant state. If these microorganisms are crucial taxa that regulate microbial symbiotic networks in water or sediment, changes or reductions in their population could hasten the collapse of such networks [8,9,10]. The enhancement of microbial proliferation in aquatic ecosystems and the inhibition of fish immune responses have been recognized as precursors to diverse bacterial pathologies [11,12,13,14]. Various bacterial infections affect fish species, including *Aeromonas salmonicida*, *Pseudomonas anguilliseptica*, *Vibrio harveyi* and *V. anguillarum*, *Moritella viscosa*, *Tenacibaculosis*, and *Lactococcus garvieae*. *Vibrio* and *Mycobacterium* are significant fish pathogens that can lead to a fish mortality rate of up to 50% and affect all the developmental stages of fish [6,15]. Although the majority of disease outbreaks are due to opportunistic bacteria that target stressed, vulnerable fish, more serious infections could also occur in the absence of environmental stressors [3,16]. Thus, the emergence of opportunistic pathogens can be attributed to a range of stressful conditions: the interplays between the host, the pathogens, and the specific environment in which they are established. A thorough comprehension of the diverse factors in the aquaculture setting is imperative for its productivity.

Antibiotic therapy has been extensively employed for years to address bacterial illnesses in aquaculture [17]. Despite being frequently utilized to prevent and manage diseases and increase cultured organism growth, only approximately 25% to 75% of antibiotics are retained by treated fish. The remaining antibiotics are excreted as unchanged compounds or their metabolites in feces and urine, contributing to the widespread accumulation of antibiotic products in open-water mariculture systems [18,19,20]. The concentration of antibiotics in brackish water (31 compounds) exceeded that in freshwater (22 compounds), indicating higher pollution levels in seawater than in freshwater [4]. This lingering presence of antibiotics could create selective pressure, contributing to the emergence of resistant bacteria [7]. Compared with susceptible strains, resistant bacteria under such antibiotic pressure are more likely to access greater nutrients and space, thereby increasing the risk of antibiotic resistance [21,22,23]. Moreover, antibiotic resistance genes (ARGs) can persist in the environment for extended periods; high concentrations have been shown to promote resistance development in intestinal bacteria, with the subsequent attachment of ARGs to human pathogens occurring via horizontal gene transfer, resulting in human infections [4,24,25,26,27]. However, the interactions of ARGs with diverse microbial communities in aquaculture systems remain largely unexplored.

*Oplegnathus punctatus* (*O. punctatus*) is a crucial commercial fish species, and China contributes to approximately 65% of the global *O. punctatus* production [15,28]. Nevertheless, the swift advancement of the aquaculture sector in Laizhou Bay has resulted in resource and environmental limitations [29,30]. An example of this is the occurrence of large-scale anoxic events and harmful algal blooms due to elevated nutrient loads or eutrophication, which has negatively impacted their culture in recent years [6,31,32]. Therefore, achieving a comprehensive understanding of the environmental variability associated with *O. punctatus* net-pen aquaculture is crucial for successful large-scale captive aquaculture management. An in-depth analysis of the impact of mariculture on the ecological environment, reflecting the distribution of microbial communities and ARGs in both seawater and sediments, will serve as a foundation for addressing the dual ecological and economic needs of mariculture.

## 2. Materials and Methods

### 2.1. Study Area and Sample Collection

The study site is in Laizhou Bay, a region in the southern Bohai Sea, covering approximately 7000 km^2^ with an average depth of around 9 m [33]. The selected mariculture area is managed under an engineered fence mode (with a circumference of 400 m and a breeding water body of 160,000 m^3^). Due to the climate in this region, the seawater is only suitable for the growth of *O. punctatus* during the annual breeding period from June to October. Consequently, the sampling in this study was scheduled to coincide with this breeding period [34]. For sampling purposes, three sites within the mariculture area (120.05° N, 37.50° E) were selected, along with three additional fixed sites in the non-aquaculture areas for comparison (CS1: 119.89° N, 37.40° E; CS2: 122.17° N, 38.99° E; and CS3: 122.51° N, 39.24° E). Sampling was carried out between June and November 2022. Water samples were taken by collecting 8 L of seawater from two different depths: the surface layer (approximately 2 m) and a layer located between 8 and 10 m above the bottom of the water column. The sampling occurred twice a month from June to August and monthly from September to November using Niskin bottles, resulting in a total of 96 seawater samples. In addition, a 5 kg sediment sample was collected once per month at each sampling site, except for two sampling events in early July and mid-July, and the sample collection tools were used to conduct seafloor operations via grapple-type mud harvesters. (total 34 sediment samples). In situ temperature, salinity, dissolved oxygen (DO), pH, pressure, and other physical and chemical properties were measured via a YSI multiparameter water quality meter (ProQuatro, Yellow Springs, OH, USA). The YSI sensors do not require temperature calibration, whereas the DO sensor was calibrated to YSI specifications at 100% saturation, while the pH meter was calibrated with buffer solutions of pH 7 and 10. The seawater that was gathered was passed through a sterile membrane with a pore size of 0.2 μm. Subsamples were then taken and placed in 50 mL polycarbonate centrifuge tubes, which were stored at −80 °C for future DNA extraction.

### 2.2. DNA Extraction and Metagenomic Sequencing

Microbial DNA extraction occurred from the sediment and seawater samples via the E.Z.N.A.^®^ Stool DNA Kit (Omega Biotek, Norcross, GA, USA) following the manufacturer’s instructions. Metagenomic shotgun sequencing libraries were created and sequenced at Shanghai Biozeron Biological Technology Co., Ltd. (Shanghai, China). Specifically, for each sample, 1 μg of genomic DNA was fragmented using a Covaris S220 focused ultrasonicator (Woburn, MA, USA), resulting in sequencing libraries with an average fragment size of about 450 bp. In this study, we focused on the high-throughput sequencing of the gene 27F-1492R region of microbial communities to assess changes in microbial diversity under different environmental conditions. To achieve this goal, we used the following primer sequences for PCR amplification: forward primer: AGRGTTYGATYMTGGCTCAG (corresponding to the start of the 27F region); reverse primer: RGYTACCTTGTTACGACTT (corresponds to the start of the 1492R region). All the samples were sequenced using an Illumina NovaSeq 6000 instrument in the paired-end 150 bp (PE150) mode. The raw sequence reads were processed with Trimmomatic v0.36 (http://www.usadellab.org/cms/uploads/supplementary/Trimmomatic, accessed on 13 August 2024) to eliminate adaptor contaminants and low-quality reads. The reads that passed quality control were then aligned to the human genome (version: hg 38) via the BWA mem algorithm (parameters: -M -k 32 -t 16, http://bio-bwa.sourceforge.net/bwa.shtml, accessed on 13 August 2024). The reads that had host-genome contamination and low-quality data removed were termed clean reads and were utilized for the subsequent analysis.

### 2.3. Read-Based Phylogenetic Annotation

Kraken2 was utilized to identify the taxonomy of the clean reads for each sample via a tailored Kraken database (minikraken_8GB_20200312). The reads were categorized into seven phylogenetic levels (domain, phylum, class, order, family, genus, and species) or were left unclassified. Taxa abundances were estimated using Bracken (https://ccb.jhu.edu/software/bracken/, accessed on 13 August 2024), which can accurately assess species- and genus-level abundances, even among closely related species. The relative abundance at a specific level in the article represents the total abundance of species within that level.

### 2.4. Metagenomic De Novo Assembly, Gene Prediction, and Gene Abundance

Clean sequence reads were produced from a collection of contigs for each sample using MegaHit (v1.1.1-2-g 02102e1) with the parameters set to “--min-contig-len 500”. The open reading frames (ORFs) of the assembled contigs were identified using METAProdigal (v2.6.3), and all the ORFs were grouped using CD-HIT [35] with the following parameters: -n 9 -c 0.95 -G 0 -M 0 -d 0 -aS 0.9 -r 0 -T 80, resulting in a unique gene set. The longest sequence from each cluster was designated as the representative sequence for each gene in this unique gene set. High-quality reads from each sample were aligned to the unique gene set via BWA-MEM (v.0.7.17). We calculated gene abundance profiles, ensuring the alignment length was ≥50 bp and sequence identity exceeded 95%. We expressed the results in transcripts per million (TPM), adjusting for gene length differences and the number of mapped reads per sample. The formula for TPM is as follows:

$$TPM=\frac{Ng}{Lg}\times \frac{1}{\sum j\frac{Nj}{Lj}}\times 10^{6}$$

In this context, Ng refers to the read count, which is the average number of reads aligned to the g gene, while Lg denotes the gene length, or the total number of nucleotides in the g gene. The index j signifies the collection of all the genes identified in a catalog, with g serving as an index for a specific gene.

### 2.5. Gene Function Annotation Based on Unique Genes

The distinct gene set was analyzed using Kofam (v1.2.0) to search the KEGG databases for proteins and obtain their functional annotations. Additionally, the unique gene set was compared to the eggNOG (v5.0) database through BLASTP searches against the NCBI NR database using DIAMOND (v0.9.22.123). Furthermore, the unique gene set was also compared with the SARG v2.3 database using DIAMOND (v0.9.22.123) BLASTP (http://blast.ncbi.nlm.nih.gov/Blast.cgi, accessed on 13 August 2024) focusing on matches with ≥80% identity and ≥70% coverage to identify ARG-like open reading frames (ORFs).

### 2.6. Data Statistics and Visualization

Initial calculations, Spearman correlation, and Wilcoxon test analyses were performed using Excel 2019 (Microsoft, Redmond, WA, USA), SPSS 25.0 (IBM, Armonk, NY, USA), and R 4.1.2 statistical software. Bar charts, scatter plots, pie charts, and Mantel test box plots were generated using the website https://www.chiplot.online/ (accessed on 13 August 2024). R 4.1.2 was also utilized for Pheatmap, canonical correlation analysis (CCA), trend analysis, the statistical analysis of metagenomic profiles (STAMP), and linear discriminant analysis effect size analysis (LEfSe). The *p*-value obtained from the Permutation Multivariate Analysis of Variance (PERMANOVA) test was less than 0.05, indicating a significant relationship between environmental factors and microbial structure.

## 3. Results

### 3.1. Environmental Factors

The measurements obtained for environmental parameters in Laizhou Bay from June to November 2022 revealed complex patterns of temporal variation and dynamic trends, along with significant seasonal differences (Figure S1, Table S1). The DO content, ranging from 2.17 to 6.71 mg/L, exhibited a temporally increasing trend with significant seasonal variation between June and November, with the highest concentrations recorded in autumn (Figure S1a). In contrast, there was no significant trend in the water pH, which ranged from 7.77 to 8.41 during the monitoring period. However, the pH values in the aquaculture areas fluctuated to a lesser extent than those in the non-aquaculture areas (Figure S1b). The water pressure in the surveyed area generally ranged between 736.90 and 756.27 mmHg, with the values increasing over time (Figure S1c). Similarly, the temperature exhibited significant seasonality, ranging from 14.57 to 28.53 °C, and tended to be negatively correlated with pressure. Salinity (22.91 to 29.56 ppt), suspended particle concentration (SPC) (36.18 to 45.73 ms/cm), and total dissolved solids (TDSs) (23.53 to 29.73 g/L) exhibited decreasing trends, with the lowest values recorded in October (Figure S1).

### 3.2. Microbial Community Composition

**Figure 1:**
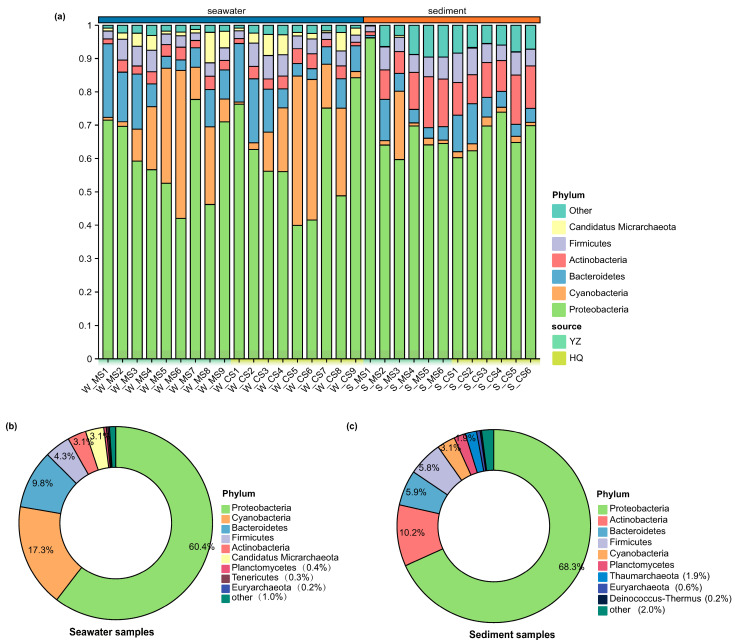
The condition of microbial communities in various environments. The proportion of microbial communities categorized by phylum (**a**), for which the top 10 most abundant taxa in seawater (**b**) and sediment (**c**) are represented by pie charts. Abbreviations: MS: mariculture area; CS: control seawater area.

In all the samples, 51 different bacterial phyla were detected. The most abundant phyla included Proteobacteria (comprising 60.43% to 68.27% of the total species), Cyanobacteria (17.28% to 3.05%), Bacteroidetes (5.93% to 9.84%), Actinobacteria (12.30% to 15.66%), and Firmicutes (4.30% to 5.82%) (Figure 1a). Proteobacteria predominated in all the samples among the 10 most abundant phyla. Additionally, Candidatus Micrarchaecta (3.08%) and Tenericutes (0.29%) were identified as bacteria unique to seawater, whereas Thaumarchaeota (1.89%) and Deinococcus-Thermus (0.24%) were detected exclusively in sediment samples. Furthermore, substantial differences in the abundances of certain taxa were observed between seawater (Figure 1b) and sediments (Figure 1c). For example, the proportions of Cyanobacteria and Actinomycetes in seawater and sediment were 17.28%, 3.09%, and 3.05%, 10.24%, respectively. Microbial community diversity in both aquaculture and open sea areas (Figure S2a) began to increase from mid-to-late June and was thereafter maintained at an elevated level for approximately 2 months. The levels began to decrease in August, along with corresponding shifts in the overall number of species. The initial collection of sediments was carried out one month after *O. punctatus* had been released for aquaculture. At that time, the sediments (Figure S2b) in the farmed area already contained the residual traces of the farmed organisms. Compared with those in the non-aquaculture area, the variety of microbial communities and the number of species in the aquaculture regions were initially low at the beginning of the survey period but reached a stable state within approximately 2 weeks. In contrast, the species diversity in the nonculture areas showed minimal changes.

**Figure 2:**
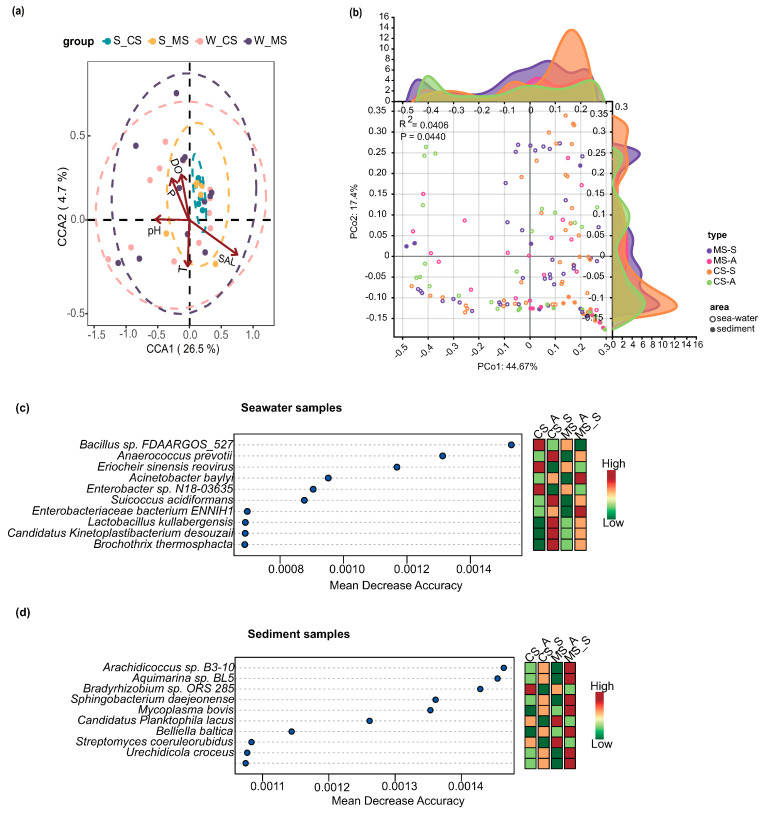
CCA of the environmental factors and bacterial communities revealed the clustering of the microbial communities in seawater and sediment influenced by environmental factors in the aquaculture and non-aquaculture environments (**a**). PCoA was performed to assess differences in community clustering and changes in density (**b**) according to different seasons; the closer the distances between the samples, the more similar are the abundances of the major species in the two communities, and vice versa. Random forest analyses were performed to screen the 15 microbial species with the highest accuracy for predicting seawater (**c**) and sediment (**d**) grouping to determine the influence of regional conditions and environmental factors on the microbial community as a whole. Abbreviations: W-MS: mariculture area; W-CS: control seawater area; S-MS: mariculture sediment area; S-CS: control sea sediment area.

**Figure 3:**
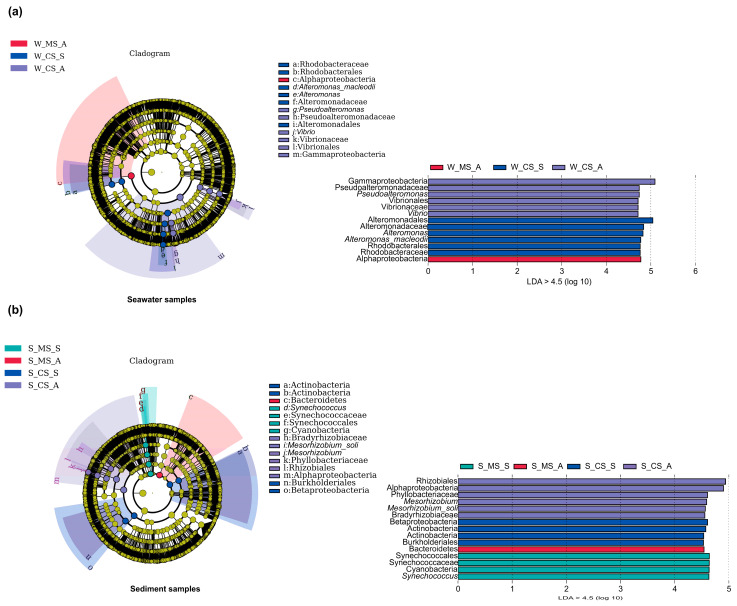
Using LEfSe analysis, the largest taxa (LDA ≥ 4.5) at the seawater (**a**) and sediment (**b**) levels were identified between the mariculture (MS) and control seawater (CS) areas. Differences were presented to the best annotated taxonomic rank. Abbreviations: MS-S: summer mariculture area; MS-A: autumn mariculture area; CS-S: summer non-aquaculture area; CS-A: autumn non-aquaculture area.

The PCoA plots demonstrate the changes in the space and time of microbial communities in both the cultured and uncultured settings (Figure 2b). They demonstrate that aquaculture impacts the microbial communities in the seawater and sediment areas to different extents. These effects are especially significant during the summer season. Regarding the microbial community detected in seawater (Figure 3a), we identified 13 biomarkers, among which Alphaproteobacteria were detected in the aquaculture areas, and 6 were detected in both summer and fall in the non-aquaculture areas. Similarly, in the aquaculture regions, four biomarkers were found in the summer and one in the autumn (Figure 3b), while in the non-aquaculture areas, four biomarkers were identified in the summer and six in the autumn. Among these, we identified the marker organisms *Alphaproteobacterium* HIMB59 and Photobacterium damselae as being more abundant in the mariculture area samples, whereas *Sulfitobacter* sp. SK025, *Alteromonas macleodii*, *Vibrio cyclitrophicus*, and *Vibrio harveyi* were found to be more abundant in the open-water areas. In the sediment samples, *Synechococcus* sp. WH 7803, Candidatus *Pelagibacter* sp. HIMB1321, *Vibrio alfacsensis*, *Vibrio harveyi*, and *Vibrio owensii* were found to be more abundant in the mariculture sediments, whereas *Mycolicibacterium aubagnense*, *Bradyrhizobium* sp. 58S1, *Bradyrhizobium* sp. 6 (2017), *Bradyrhizobium* sp. SK17, and *Mesorhizobium soli* were more abundant in the open-water sediments.

### 3.3. Relationships between Bacteria and Environmental Factors

**Figure 4:**
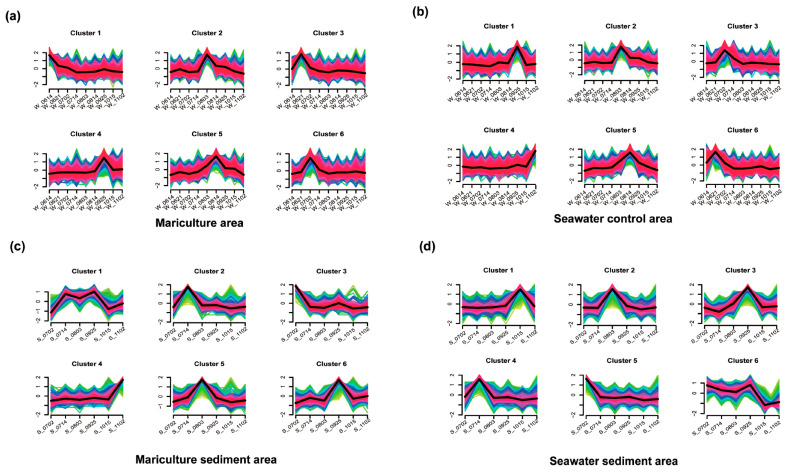
Model profiles were used to analyze the patterns of change in the relative abundance of species in the seawater and sediment areas, respectively. Each trend represents the abundance change profile of a model (**a**–**d**).

Based on the analysis of species abundance in the environmental microbial communities across different regions (Figure 4 and Figure S4), this study revealed varying microbial abundances and trends in each stratum. Among these, Group 5, which represented the highest number of annotated species (2160 species) in the mariculture zone, presented an initial increase in the number of microorganisms, followed by a decrease and a peak in abundance in mid-August. Similarly, the most abundant annotated species in seawater from the non-aquaculture areas (2000 species) also exhibited a Group 5 pattern, which was consistent with patterns observed in the mariculture zones.

Combined with the biomarkers obtained from the LEfSe analysis in seawater (Figure 3a), the Alphaproteobacteria markers were prominently represented in the seawater of aquaculture zones, and in conjunction with Figure 4a, it is known that they were located in Cluster 2, maintaining the same cluster changes as the 1221 microorganisms in this trend (Figure S3). Among the markers detected in the control seawater zone, those observed in this study were Gammaproteobacteria, Vibrionales, Alteromonadales, and Rhodobacterales, which were primarily distributed throughout Clusters 1 and 4 (Figure 4b). The abundance of the relevant microorganisms in the previous period revealed that the structure of the associated microorganisms was stable in the initial period, with a clear peak occurring between September and October. Five marker species were detected in the sediment samples collected in the cultivation zone (Figure 3b), represented by Bacteroidetes and Synechococcaceae, and were present mainly in Clusters 1 and 5 (Figure 4c). In the final sediment of the nonincubation zone, 10 marker species, such as Actinobacteria and Mesorhizobium, were concentrated mainly in Clusters 3 and 6 (Figure 4d), with the month of September being the period with the highest abundance, resulting in a significant increase in flora, accounting for 57.73% of the total number of microorganisms involved in this period (Figure S3).

**Figure 5:**
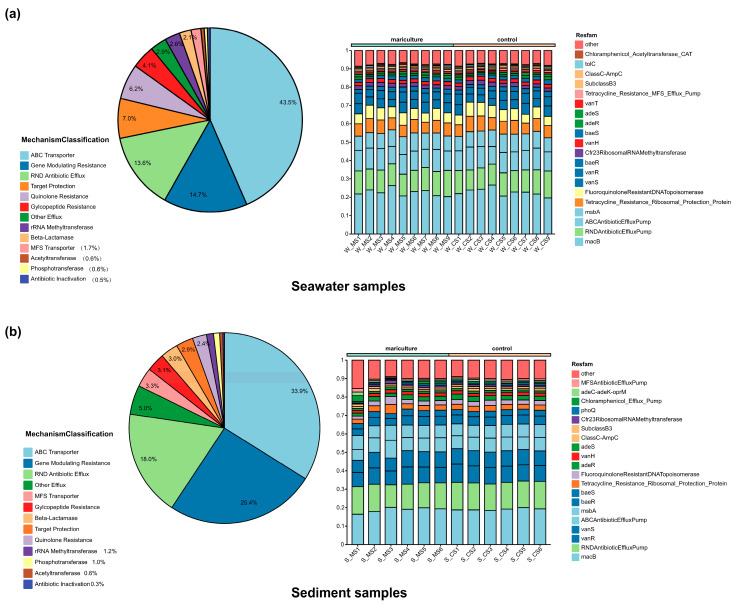
Features and distinctions of the antibiotic resistance genes within the broader aquaculture setting, mechanism of action (pie), and relative abundance (bar) of the antibiotic resistance genes in seawater (**a**) and sediment (**b**) during the period from June and November 2022.

The clusters observed with the LEfSe markers could provide further insights into the variations among the microorganisms in specific regions. In the mariculture areas (Figure 3a), Clusters 3 and 6 both showed initial rapid increases in June, followed by a peak and subsequent decline before the end of July (Figure 5a). However, these clusters differed in that the initial rise in Cluster 3 began earlier in the year than that of Cluster 6 and returned to the initial level at an earlier stage. We speculated that this could be associated with the stronger adaptive mechanisms of the microorganisms following this pattern. In the sediment collected in the culture areas, Cluster 3 clearly exhibited downward clusters from June to July before reaching a steady state, whereas Cluster 5 reached a single peak in August (Figure 4c). The findings of our CCA indicated that in each of the four assessed environments (Figure 2a), salinity had a significant effect on the overall change in microbial growth. Moreover, the sediment of the non-aquaculture areas was characterized by the smallest accumulation of microorganisms, providing further evidence that the mariculture of *O. punctatus* could contribute to enhancing the microbial diversity in sediments to a certain extent, although the corresponding effect in seawater was less pronounced. Based on random forest analysis, we identified 15 features for determining the accuracy of sample group prediction, with *Clostridium pasteurianum*, *Synechococcus phage S-CAM 4*, *Leptotrichia wadei*, and *Salmonella phage NR01* being identified as more prominent species in seawater (Figure 2c), whereas the more prominent species in sediments (Figure 2d) were *Campylobacter insulaenigrae*, *Mycoplasma bovoculi*, and *Prolixibacteraceae bacterium* WC007.

### 3.4. Occurrence of Antibiotics in Mariculture

Seawater was classified into 13 resistance mechanisms and 92 resfam categories. Among the main resistance mechanisms in seawater (Figure 5a), ABC transporters accounted for 43.5%, gene-modulating resistance accounted for 14.7%, RND antibiotic efflux accounted for 13.58%, target protection accounted for 6.99%, and quinolone resistance accounted for 6.18%. The top 10 genes (Figure 5a) included macB, the RND antibiotic efflux pump, antibiotic efflux pump, the ABC antibiotic efflux pump, msbA, the tetracycline resistance ribosomal protection protein, fluoroquinolone-resistant DNA topoisomerase, and others. The resistance mechanisms identified in the sediment samples revealed notable discrepancies when contrasted with those present in the seawater samples. In the sediments (Figure 5b), the ABC transporter accounted for 31.11%, the gene-modulating resistance accounted for 23.61%, the RND antibiotic efflux accounted for 19.68%, the other efflux accounted for 6.5%, and the MFST transporter accounted for 5.36%. The top 10 results (Figure 5b) for the sediment samples included macB, RND antibiotic efflux pump, vanR, vanS, ABC antibiotic efflux pump, msbA, baeR, baeS, tetracycline resistance ribosomal protection protein, and fluoroquinolone-resistant DNA topoisomerase. Notably, the proportion of genes associated with gene-modulating resistance (vanR, vanS, baeR, and baeS) in the sediment was significantly greater than that in seawater, whereas reductions in quinolone-resistant DNA topoisomerase (fluoroquinolone-resistant DNA topoisomerase), methyltransferase ribosomal RNA methyltransferase (Cfr23 ribosomal RNA methyltransferase), and target protection (tetracycline-resistant ribosomal protection protein) were detected.

**Figure 6:**
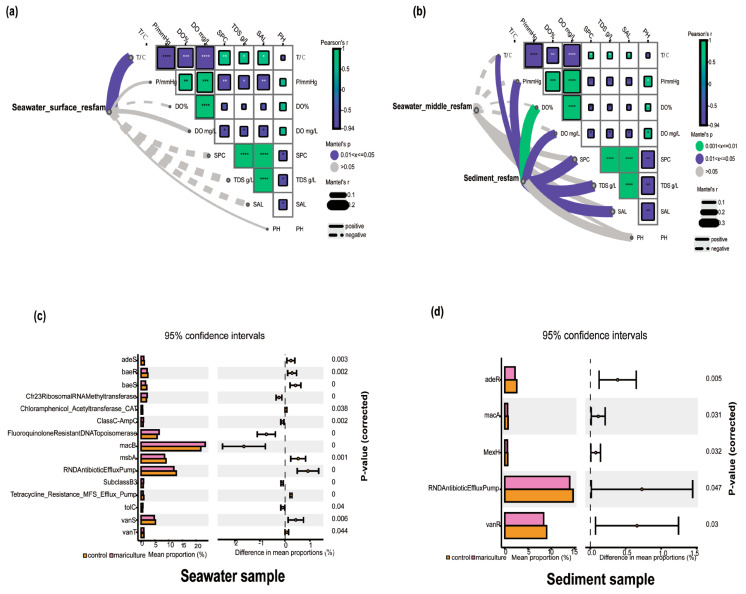
A correlation analysis was conducted to examine the relationship between antibiotic resistance genes and environmental factors in the surface (**a**), mid-water, and sediment (**b**) levels of seawater utilizing the Mantel test. The lower left figure displays the pairwise comparisons of environmental factors, with a color gradient illustrating Spearman’s correlation coefficients. The thickness of the lines represents the partial Mantel’s r statistic for the respective correlations, with thicker lines indicating stronger correlations. Additionally, antibiotic resistance genes in seawater (**c**) and sediment (**d**) were screened using stamp analysis.

The Mantel test, which assesses ongoing changes in ARG abundance, revealed that temperature had the most significant effect on the ARGs detected in surface water (Figure 6a), with a positive correlation trend. For the water samples from the middle levels of the water column (Figure 6b), no notable environmental factors affecting ARGs were identified, but the factors positively correlated with the ARGs in surface water were the opposite at medium depths. In comparison, the ARGs detected in the sediments were more strongly influenced by environmental factors, all of which had positive effects. Among these factors, dissolved oxygen was notably significant, reflecting its importance in the survival of microorganisms in sediments and its role in determining microbial diversity and functional composition. With respect to the impact of aquaculture on ARGs, the STAMP analysis revealed significant effects (*p* < 0.05) of *O. punctatus* mariculture on the proliferation of Fluoroquinolone-Resistant DNA Topoisomerase and ClassC-AmpC in seawater (Figure 6c), as well as a significant inhibition (*p* < 0.05) of adeS, baeR, and msbA. In the sediments (Figure 6d), mariculture appeared to significantly inhibit the proliferation of adeR (*p* ≤ 0.05).

### 3.5. Associations between Antibiotic Resistance Genes and Microbiomes

**Figure 7:**
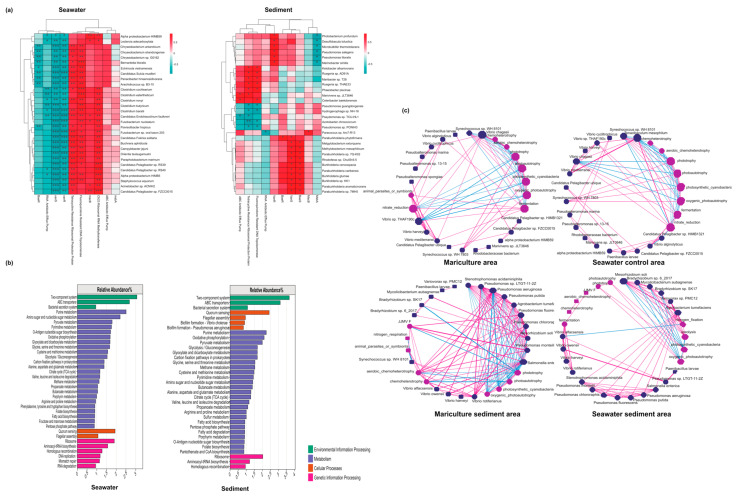
Responses of the microbial community with respect to functional genes, showing the correlation between the dominant microorganisms in seawater and sediment and the antibiotic resistance genes of the top 10 species (Single Asterisk (*): Indicates statistical significance at the 5% level (*p* < 0.05).Double Asterisks (**): Indicates statistical significance at the 1% level (*p* < 0.01).Triple Asterisks (***): Indicates statistical significance at the 0.1% level (*p* < 0.001).(**a**), and KEGG secondary pathway annotation functional genes detected in seawater and sediment (**b**). The network structure analysis of microorganisms and related microbial functions predicted by faprotax in seawater and sediment in the cultured and non-aquaculture areas (significantly related generated lines) (**c**). The correlation coefficient threshold was set at 0.7 (*p* < 0.5). The top 30 bacteria and predicted bacterial functions are denoted by dark purple and light purple, respectively. The blue and pink lines indicate negative and positive correlation, respectively, and the width of the lines reflects the importance of the correlation (*p*-value).

Based on our analysis of the correlations between the top ten most abundant ARGs annotated in the region and the microbial species in the region, we identified the top 30 dominant microorganisms (with a correlation dilution threshold of 0.7) (Figure 7a). In the seawater of the mariculture area, we observed a highly significant positive correlation between tetracycline resistance ribosomal protection protein, fluoroquinolone-resistant DNA Topoisomerase, and Cfr23Ribosomal RNA methyltransferase and dominant microorganisms. Conversely, vanR, vanS, RND antibiotic efflux pump, and baeR showed a negative correlation, with vanR and vanS exhibiting extremely significant negative correlations. In the sediment of the mariculture area, baeS and vanS were significantly positively correlated with *Paraburkholderia phytofirmans*, *Methylorubrum extorquens*, *Methylobacterium mesophilicum*, *Paraburkholderia* sp. 7Q-K02, *Rhodoferax* sp. CHu59-6-5, *Burkholderia cenocepacia*, *Paraburkholderia caribensis*, *Burkholderia glumae*, *Burkholderia* sp. KK1, *Paraburkholderia aromaticivorans*, and *Paraburkholderia* sp. 7MH5; whereas vanR was significantly positively correlated with *Photobacterium profundum*, *Desulfobacula toluolica*, *Microbulbifer thermotolerans*, *Pseudomonas salegens*, *Pseudomonas litoralis*, and *Marinobacter similis*. Additionally, *tetracycline resistance ribosomal protection protein*, *fluoroquinolone-Resistant DNA Topoisomerase*, and the ABC *antibiotic efflux pump* were significantly positively correlated with *Ketobacter alkanivorans*, *Ruegeria* sp. AD91A, *Maribacter* sp. T28, *Ruegeria* sp. THAF33, *Phaeobacter piscinae*, *Marivivens* sp. JLT3646, and *Celeribacter baekdonensis* and were also associated with *Pseudomonas guangdongensis*, *Hydrogenophaga* sp. nH-16, *Pseudomonas* sp. TKU-HL1, *Azotobacter chroococcum*, and *Pseudomonas*, whereas PONIH3 and msbA were negatively correlated with specific species.

The results of the KEGG metabolic pathway annotation at the top 50% level_2 for seawater and sediment revealed that the identified genes were primarily concentrated in pathways related to environmental information processing, cellular functions, metabolism, and the processing of genetic information (Figure 7b). In the seawater samples, the two-component system had the highest proportion of a single pathway (3.68%), followed by ABC transporters (3.11%). The corresponding proportions of these two categories in the sediment samples were 3.06% and 2.68%, respectively. These findings indicate a slightly greater functional enrichment of metabolism and greater diversity. In both seawater and sediments, purine metabolism was found to be particularly prominent, whereas oxidative phosphorylation appeared to be more pronounced in sediments and the metabolism of amino sugars and nucleotide sugars was more significant in seawater. It is important to highlight that the amount of genetic information processing found in the sediment samples was considerably less than what was observed in seawater, whereas the proportion of the quorum-sensing domain of cellular processes was significantly greater.

Having initially used the FAPROTAX software (version 1.2.6) [36] to predict the functional abundance of the samples based on the microbial abundance of the samples, we conducted a network analysis to examine the correlations between the microorganisms and functions (Table S2). We obtained the values of 48 and 28 for the maximum positive and negative correlations, respectively, in the seawater of the mariculture areas. The maximum value for the degree of centrality was 11 (Table S3 and Figure 7c). Furthermore, we obtained the closeness centrality values of 14.83 and 14.67 for Vibrio chagasii and nitrate reduction, respectively. Vibrio chagasii was found to have the highest betweenness centrality value of 106.66 (Table S2). For the seawater in the non-aquaculture areas, we obtained the maximum positive and negative correlation values of 46 and 31, respectively, and a value of 11 for the maximum degree of centrality (Table S4).

For the sediment samples from the mariculture areas (Figure 7c), the correlation network revealed the maximum positive and negative correlation values of 95 and 46, respectively (Table S5). The maximum degree of centrality value of 19 was observed for Salmonella enterica, with a closeness centrality of 22. The highest betweenness centrality value (84.67) was obtained for Mesorhizobium soli (Table S2). In comparison, the sediments from the non-aquaculture areas presented positive correlation values of up to 68 and negative correlation values of up to 11. Mesorhizobium soli presented the highest values for degree of centrality, closeness centrality, and betweenness centrality at 9, 16.17, and 308.4, respectively. Notably, betweenness centrality values of up to 290 and 266 were recorded for nitrogen fixation and Agrobacterium tumefaciens, respectively (Table S2). Furthermore, significant reductions in the proportion of Tenacibaculum mesophilum and notable increases in functional animal parasites or symbionts were detected in the seawater of some mariculture areas compared with those in the non-aquaculture areas. Additionally, compared with the sediments from the non-aquaculture areas (Table S6), there was a general increase in bacterial abundance in the sediments collected from the mariculture areas, including populations of Salmonella enterica, Pseudomonas aeruginosa, Pseudomonas fluorescens, and Pseudomonas chlororaphis, among others. Significant increases in the predicted microbial functions related to animal parasites or symbionts, photosynthetic cyanobacteria, and oxygenic photoautotrophic respiration were also detected. In contrast, reductions were noted in ureolysis, nitrogen fixation, and fermentation. The analysis of the varying impacts of mariculture on seawater and sediments indicates that microorganisms and their functions have a significantly less pronounced influence on seawater than on sediment, as indicated by the values obtained for degree, betweenness, and closeness centralities. Furthermore, the correlations among the different levels of sediment in the mariculture areas were also relatively close, thereby enhancing the stability of the ecological structure in this region.

## 4. Discussion

Microbial responses in different environments were influenced by various external factors. This research identified Proteobacteria, Cyanobacteria, Bacteroidetes, and Firmicutes as the main bacterial phyla present in the seawater samples taken from the mariculture regions. Additionally, Verrucoi microbia, which was typically present in soil, water, and animal intestines, was highly abundant in the sediments from these regions, indicating a strong association with the cultured organisms [37]. Additionally, the main phyla found in the sediments were identified as Proteobacteria, Bacteroidetes, Actinobacteria, and Firmicutes. The various diversity indices, uniformities, and correlations indicate that the relationships between microorganisms in sediment and their predicted functions are more intricate than those in water [9]. Furthermore, the mutual interactions among the species in water were more easily disrupted by the loss of a single species than those in sediments. This diversity promotes the stability of microbial communities [38]. For example, bacteria in the phyla Acidobacteria, Firmicutes, and Actinobacteria play vital roles in breaking down nutrients and are crucial species in the composition of microbial networks prevalent in wastewater treatment systems [39]. Similarly, bacteria in the phylum Bacteroidetes aid in breaking down polymers and intricate organic substances, such as decomposing deceased cells with polysaccharides and proteins [40]. This leads to the creation of fundamental organic substances, like ethanol and lactic acid, that can be used by different organisms [41,42]. Furthermore, the key species was *Synechococcus* sp. WH 8101 (Cyanobacteria), which utilizes inorganic nutrients for growth through photosynthesis [43]. As it descends to lower water column levels or sediments, its buoyancy increases as nutrients are replenished. This allows cells to return to the surface layers of the water column [44,45,46]. These characteristics were conducive to maintaining a high abundance of cyanobacteria [44]. The degradation of antibiotics tends to be more rapid under aerobic conditions than under anaerobic conditions [47]. Consuming oxidants, substrate components in mariculture environments, and factors like dissolved organic matter (DOM) and ammonia may affect the effectiveness of antibiotic oxidation [48,49]. Consequently, the sediments of water bodies were the primary sites for the accumulation of residual antibiotics, which, in turn, promoted the proliferation of ARGs among the sediment microbial populations.

This research revealed strong positive relationships between bacteria from the phyla Proteobacteria, Actinobacteria, and Cyanobacteria and various ARGs in an aquaculture environment, including both seawater and sediment. These findings indicate that bacteria in these main phyla could host ARGs in maricultural environments, thereby playing a key role in the spread of these genes. This group of bacteria exhibits a wide variety, a significant level of multi-drug resistance, the presence of virulence genes, and the resistance of *Photobacterium damselae* in mariculture areas which were influenced primarily by temperature and antibiotic use [50]. Significant changes in bacterial abundance and diversity were observed during the monitoring period, particularly in June and July. The observed changes in microbial communities likely originate from the cultivation of species-specific attributes and activities that largely shape the dynamics and diversity of the microbial composition in the ecosystems where they are found. The results from the LEfSe analysis indicated that *Vibrio alfacsensis*, *Vibrio harveyi*, and *Vibrio owensii* accounted for the largest proportion of marker microorganisms in the sediments collected from the surveyed mariculture areas [51,52,53,54]. During the early phase of introducing maculatogony, there were notable fluctuations in the populations of these species, but subsequently, their numbers stabilized. These bacteria were commonly found in estuaries, bays, coastal areas, and marine life and have been identified as major bacterial pathogens in fish and shellfish [55,56]. Moreover, certain species of *Vibrio* have been identified as the causal agents of specific diseases in humans [57]. Based on our findings in this study, we speculated that introducing organisms for mariculture may, to some extent, help reduce the abundance of harmful bacteria in the environment.

Antibiotics may have notable environmental effects, and specific environmental factors under maricultural conditions could alter their characteristics. High salinity and DOM content, for example, could impact antibiotic movement and alteration, potentially fostering the spread of ARGs [58,59]. The major ARGs detected in maricultural environments belong to the sul and tet gene families. Among these genes, the tetB gene encodes an energy-dependent efflux protein found in plasmids [60,61]. ABC transport has been identified as the primary mechanism of action associated with ARGs in seawater and sediment. Additionally, certain antibiotics, such as sulfonamides, have been detected in seawater because of their relatively high hydrophobicity, whereas tetracyclines were usually found in shallow sediments because of their strong adsorptive capacity [50,62]. The long-term accumulation of residual feed, excrement, and dead organisms in mariculture areas may result in changes in the physical and chemical properties of sediment surfaces, including an accumulation of DOM, which creates conditions conducive to the degradation of sulfonamides [12,63]. This accumulation could explain the adsorption of various hydrophobic antibiotics in sediments.

A higher percentage of genes associated with metabolism was found in the sediment samples compared to the seawater samples. This result suggests changes in bacterial metabolism in response to antibiotics, supporting the identification of mutations in metabolic genes contributing to antibiotic resistance [64,65]. Antibiotics indirectly affect bacteria by altering their metabolism, potentially impacting core metabolic pathways, which represents a fundamental mechanism of antibiotic resistance [41]. Additionally, two-component systems, important transduction systems in a diverse range of microorganisms, were found to be the most abundant ARGs in all the surveyed regions [66]. For example, CpxAR, PmrAB, and BaeSR have been identified as the regulators of drug resistance in *Salmonella* species [67]. These two-component systems could interact with complex regulatory networks associated with drug resistance [68]. Compared with those in the seawater samples, the proportions of the two-component systems in the sediment samples increased significantly. Vancomycin-resistant van gene clusters and baeSR gene clusters showed regional dominance in the sediment samples [69,70]. The VanS/VanR two-component system, located in the van gene cluster, triggers the expression of van genes in response to extracellular glycopeptide antibiotics. This system has been identified in human pathogens like *Enterococcus faecalis*, *Enterococcus faecium*, and *Staphylococcus aureus*, as well as in glycopeptide-producing actinomycetes such as *Amylococcus aureus*, *Actinoplanes teichomyceticus*, and *Streptomyces toyocaensis*, and in non-glycopeptide-producing actinomycetes like *Streptomyces coelicolor* [71,72,73]. Another two-component system identified was BaeSR, linked to the extracytoplasmic stress response mechanism in Escherichia coli. It contributes to the organism’s adaptation to environmental stressors. This system primarily detects and reacts to external environmental pressures that impact the cell, including antimicrobial peptides, variations in pH, and bile salts. When the cell faces these stresses, the BaeSR system is activated and triggers adaptive responses to aid in bacterial survival and recovery [74,75,76]. By promoting the downregulated expression of the torR protein, the overexpression of baeR has been demonstrated to influence bacterial drugs through the ABC transport system, resulting in an increase in bacterial resistance to fluoroquinolones [41,77]. In addition, high levels of the Cfr23 ribosomal RNA methyltransferase were detected in seawater. CFR is an important resistance gene to which antibiotics bind to the peptidyl transferase center of ribosomes. It encodes an RNA methyltransferase, and this modification confers resistance to a range of antibiotics, including streptomycin, chloramphenicol, florfenicol, linezolid, and clindamycin [78]. Notably, this gene spreads readily through microbial communities, thus granting antibiotic resistance without any previous exposure [79,80,81]. The flow of seawater may also have contributed to the widespread dissemination of these genes.

Notably, quorum sensing, a crucial process for collective energy generation in bacteria, was significantly greater in the sediments than in seawater [82,83,84]. The specific microorganisms in the sediments presented greater cohabitation with dense connections compared with those in the water. Additionally, there was significantly greater burstiness in their interactions. Different environmental stimuli seem to drive various types of functional evolution in the same species. Quorum sensing regulates a range of characteristics, including the production of virulence factors in pathogens like *Staphylococcus aureus*, *Bacillus cereus*, *Pseudomonas aeruginosa*, *and Vibrio cholerae*, all of which have multiple quorum-sensing pathways. These bacteria utilize population-sensing mechanisms to ensure that they start expressing virulence factors only when the optimal bacterial density for infection in the host body is reached, thereby maximizing their chances of survival and spread. Examining the role of the population-sensing system in regulating virulence in pathogenic bacteria is crucial for developing new antimicrobial strategies and therapies [82,85]. These properties highlight the complex interactions among different resistance mechanisms and cross-resistance [86,87]. Therefore, investigating the associations among quorum sensing in these pathogens could contribute to the development of new antimicrobial therapies.

## 5. Conclusions

The findings of this study indicate that mariculture practices may affect the diversity and structure of microbial communities in the surrounding ecosystem. The accumulation of residual antibiotics in these environments can induce the proliferation of ARGs. Given the diverse physical and chemical properties of environments in various fields, the types of antibiotics that may accumulate also vary. This variation could be reflected in regional disparities in antibiotic resistance patterns, with functional genes responsive to the external environment being key targets. The present research revealed that compared with those in seawater, the microbial community in sediment was more diverse and structural. This complex biodiversity and interconnection among species play vital roles in maintaining ecological balance and enhancing system stability. The analysis of sediment microbial communities indicates their superior ability to adjust to environmental changes, ensuring the persistence and efficiency of ecological processes. Collectively, these findings highlight the complex and dynamic nature of the interactions between microorganisms and the environment. These interactions are characterized by regional specificity, providing comprehensive insights for establishing a more rational use of marine resources.

## Data Availability

SRA data generated in this study was uploaded to the NCBI database. Project number PRJNA1129666, PRJNA1129667, and PRJNA1139249.

## Supplementary Materials

The following supporting information can be downloaded at: https://www.mdpi.com/article/10.3390/biology13080618/s1, Figure S1. Periodic environmental factors; Figure S2. The richness and diversity of seawater (a) and sediment (b) samples between June and November were assessed based on the α-diversity index. Abbreviations: MS: mariculture area; CS: control seawater area. Figure S3. Model profiles were used to analyze the patterns of changes in the relative abundance of species in seawater and sediment environments, respectively; Figure S4. Each trend represents the abundance change profile of a model; Table S1. Basic physicochemical parameters of seawater in Laizhou Bay, Bohai Sea, China; Table S2. Correlation coefficients for regionally dominant microorganism and predicted functional structure network; Table S3. Correlation between microbial species and function in the seawater of the mariculture areas; Table S4. Correlation between microbial species and function in the seawater of the non-mariculture areas; Table S5. Correlation between microbial species and function in the sediment of the mariculture areas; Table S6. Correlation between microbial species and function in the sediment of the non-mariculture area.
