# Supplementary material for: Macrogenomics Reveals Effects on Marine Microbial Communities during Oplegnathus punctatus Enclosure Farming
Source: Biology (Basel). 2024 Aug 15;13(8):618. doi: 10.3390/biology13080618 (PMC11351227; doi:10.3390/biology13080618)
Supplement: Supplementary file 1 [file biology-13-00618-s001.zip › biology-3083949-supplementary.pdf]

Supplementary materials

Microbial community response to environmental antibiotics in the offshore aquaculture model

Lijun Wang[1†], Xiaofei Lu[1†], Zhikai Xing [1], Xindong Teng[3],Shuang Wang[1], Tianyi Liu [1], Li Zheng [3,4], Xumin Wang[1*], Jiangyong Qu[1*]

College of Life Science, Yantai University, Yantai 264005, China; wanglijun@ytu.edu.cn (L.W.); luxiaofei18@outlook.com (X.L.); xingzhk@ytu.edu.cn (Z.X.); wangshuang0456@126.com (S.W.); liutianyi0719@163.com (T.L.)

2 Qingdao International Travel Healthcare Center, Qingdao 266071, China; tengxindeng@163.com

3 First Institute of Oceanography, Ministry of Natural Resources, Qingdao 266061, China; zhengli@fio.org.cn

4 Laboratory of Marine Ecology and Environmental Science, Qingdao National Laboratory for Marine Science and Technology, Qingdao 266061, China

* Correspondence: wangxm@ytu.edu.cn (X.W.); qjy@ytu.edu.cn (J.Q.)

† These authors contributed equally to this work.

This file contains supplementary 4 figures, 6 tables.

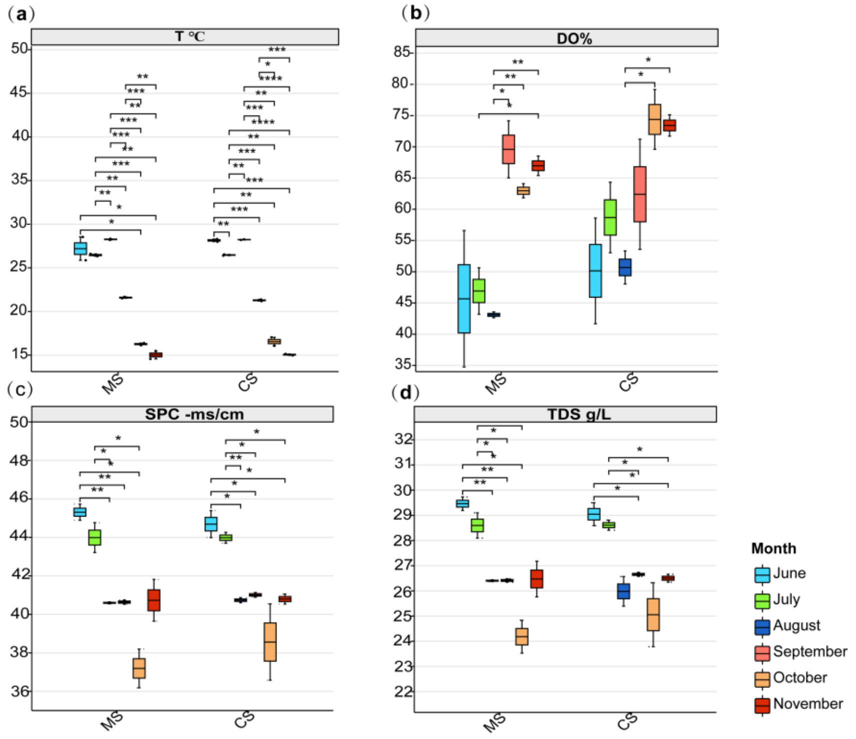

Figure S1. Periodic environmental factors. Temperature (T) (a). Dissolved oxygen percentage (DO%) (b), suspended particle concentration (SPC) (c), and total dissolved solids (TDS) (d) were recorded between June and November 2022 in the mariculture (MS) and open water (CS) areas.

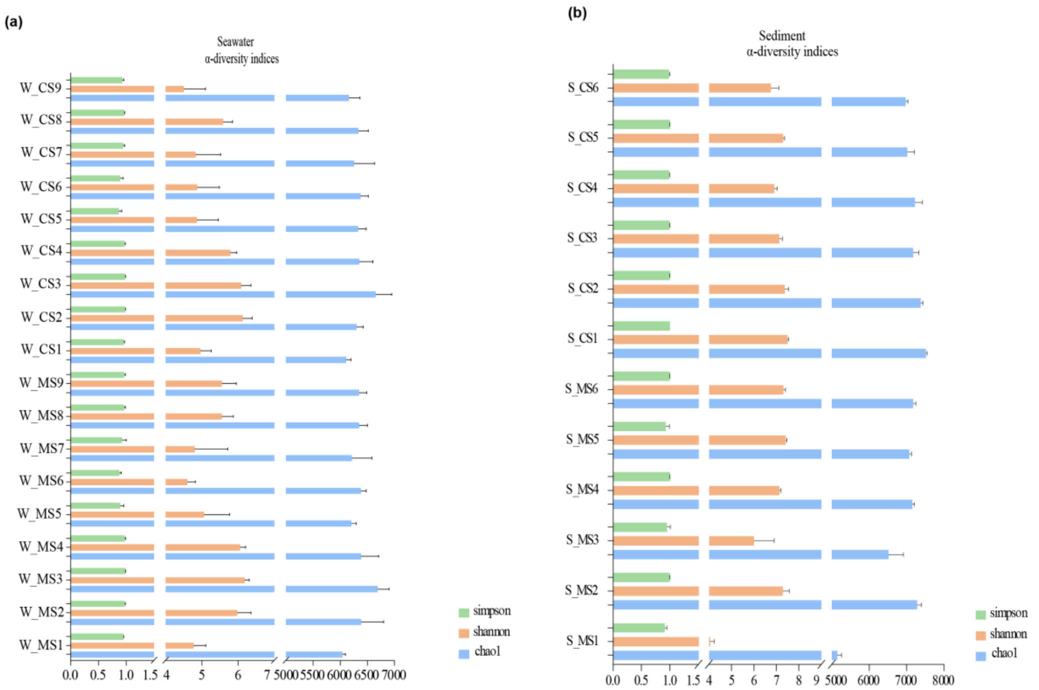

Figure S2. The richness and diversity of seawater (a) and sediment (b) samples between June and November were assessed based on the α-diversity index. Abbreviations: MS: mariculture area; CS:

control seawater area.

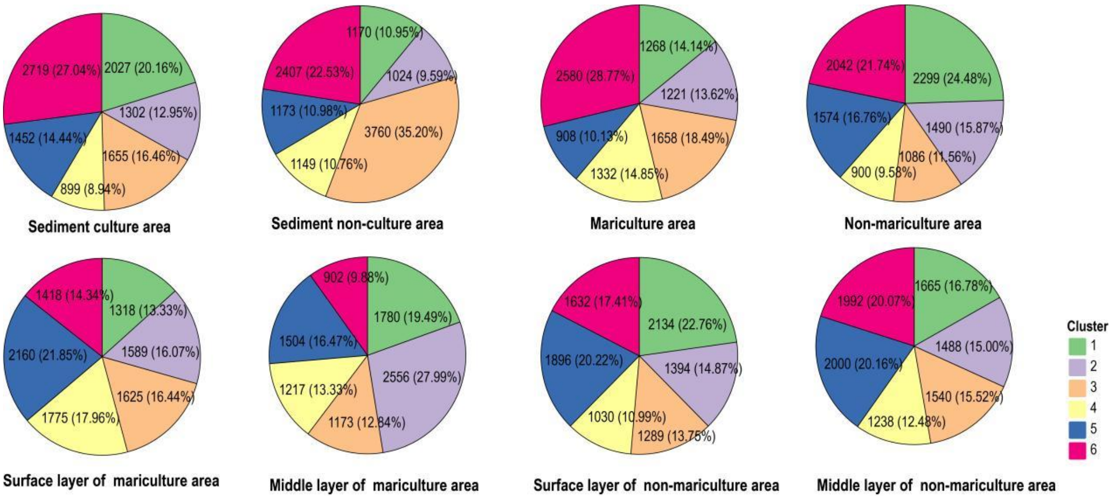

Figure S3. Model profiles were used to analyze the patterns of changes in the relative abundance of species in seawater and sediment environments, respectively. The proportion of relative species abundance contained in each cluster.

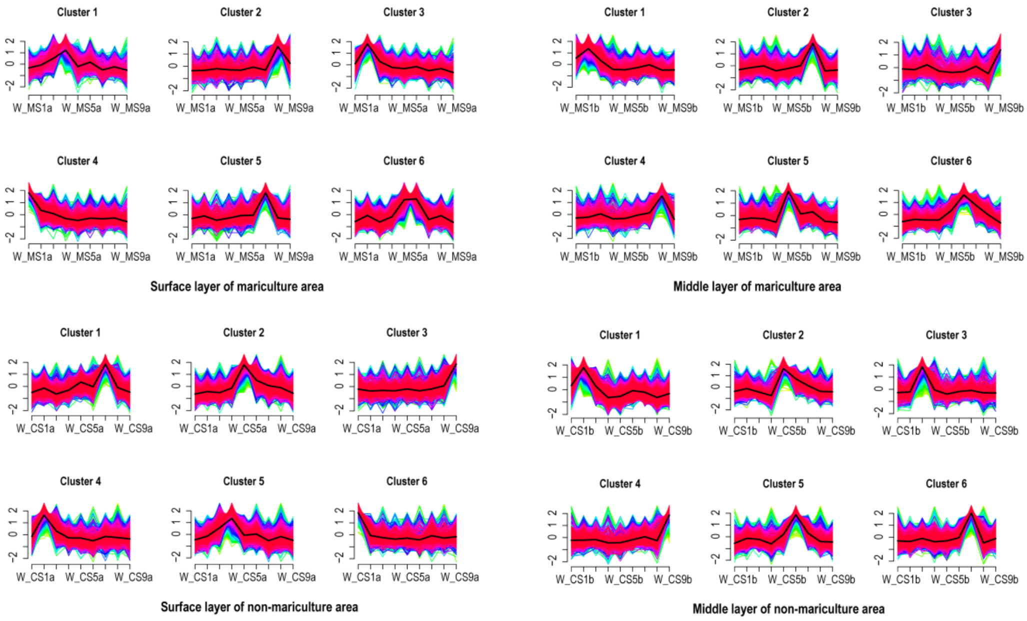

Figure S4. Each trend represents the abundance change profile of a model. Abbreviations: W-MSa: Surface layer of the water column in the mariculture area; W-MSb: Middle layer of the water column in the mariculture area; W-CSa: Surface layer of the water column in the non-mariculture area; W-CSb: Middle layer of the water column in the non-mariculture area.

Table S1. Basic physicochemical parameters of seawater in Laizhou Bay, Bohai Sea, China.

ID	T/℃	P/mmHg	DO%	DO mg/L	SPC	TDS g/L	Salinity	PH
MS_6a	25.87	738.73	56.60	3.80	44.89	29.19	29.04	7.98
MS_6b	28.53	737.33	34.73	2.17	45.73	29.73	29.56	7.77
CS_6c	28.30	736.90	58.60	3.88	45.39	29.50	29.31	7.94
CS_6d	28.00	736.90	41.68	2.80	43.99	28.59	28.38	7.83
MS_7a	26.60	746.23	50.63	3.43	43.21	28.10	27.81	7.89
MS_7b	26.33	746.30	43.20	2.83	44.77	29.10	28.92	7.93
CS_7c	26.40	746.73	53.03	3.95	44.26	28.81	28.58	8.00
CS_7d	26.53	746.87	64.33	4.35	43.70	28.41	28.16	7.99
MS_8a	28.20	741.60	42.63	2.78	40.55	26.37	25.87	8.18
MS_8b	28.33	741.53	43.57	2.89	40.65	26.44	25.96	8.21
CS_8c	28.27	741.67	53.33	3.63	40.61	25.39	25.89	8.29
CS_8d	28.20	741.23	48.03	3.18	40.87	26.57	26.09	8.13
MS_9a	21.51	752.28	65.03	4.85	40.75	26.49	26.12	8.25
MS_9b	21.68	752.23	74.16	5.52	40.53	26.34	25.96	8.24
CS_9c	21.38	752.97	71.23	5.35	40.89	26.58	26.22	8.33
CS_9d	21.19	752.83	53.58	4.35	41.14	26.74	26.41	8.33
MS_10a	16.13	755.80	64.10	5.46	36.18	23.53	22.91	8.14
MS_10b	16.38	755.73	61.83	5.15	38.20	24.83	24.34	8.20
CS_10c	16.07	755.80	79.17	6.71	36.58	23.78	23.20	8.27
CS_10d	17.03	755.80	69.60	5.45	40.54	26.32	25.90	8.09
MS_11a	14.57	756.13	68.53	5.95	39.64	25.76	25.32	8.06
MS_11b	15.47	756.20	65.40	5.54	41.82	27.18	26.87	8.08
CS_11c	14.97	756.23	75.13	6.41	40.54	26.35	25.95	8.41
CS_11d	15.13	756.27	71.70	6.12	41.06	26.66	26.27	8.34

Abbreviations: MS_a: Surface layer of mariculture area; MS_b: Middle layer of mariculture area; CS_c: Surface layer of non-mariculture area; CS_d: Middle layer of non-mariculture area.

Table S2. Correlation coefficients for regionally dominant microorganism and predicted functional structure network.

ID	Mariculture area			Marine non-aquaculture area			ID	Mariculture sediment area			Marine non-aquaculture sediment area		
	degree	Closeness Centrality	Betweenness Centrality	degree	Closeness Centrality	Betweenness Centrality		degree	Closeness Centrality	Betweenness Centrality	degree	Closeness Centrality	Betweenness Centrality
Tenacibaculum mesophilum	/	/	/	10	14.25	24.82	*Synechococcus* sp. WH 8101	4	13.50	54.59	/	/	/
Vibrio chagasii	11	14.83	106.66	9	13.75	19.93	animal_parasites_or_symbionts	4	13.33	13.83	/	/	/
nitrate_reduction	11	14.67	45.34	11	15.33	155.39	nitrogen_respiration	1	9.07	0.00	/	/	/
oxygenic_photoautotrophy	11	14.50	16.34	11	14.58	19.07	*Mesorhizobium soli*	9	16.42	84.67	9	16.17	308.40
photoautotrophy	11	14.50	16.34	11	14.58	19.07	nitrogen_fixation	/	/	/	7	14.67	290.00
photosynthetic_cyanobacteria	11	14.50	16.34	11	14.58	19.07	Agrobacterium tumefaciens	13	18.83	2.22	8	14.65	266.00
phototrophy	11	14.50	16.34	11	14.58	19.07	ureolysis	/	/	/	8	14.50	20.40
fermentation	11	14.50	29.10	10	14.25	20.39	*Bradyrhizobium* sp. SK17	4	13.75	12.83	7	14.00	14.40
Vibrio sp. THAF190c	10	14.17	31.21	6	11.75	34.21	*Mycolicibacterium aubagnense*	3	11.12	0.00	7	14.00	14.40
Synechococcus sp. WH 8101	8	12.75	2.95	9	13.58	8.85	*Bradyrhizobium* sp. 6_2017	3	11.12	0.00	7	14.00	14.40

aerobic_chemoheterotrophy	7	11.92	1.44	6	11.03	0.00	photosynthetic_cyanobacteria	13	19.17	37.38	8	13.82	46.00
Vibrio mediterranei	6	11.83	9.35	4	10.12	7.12	oxygenic_photoautotrophy	13	19.17	37.38	8	13.82	46.00
animal_parasites_or_symbionts	6	11.33	4.60	/	/	/	*Vibrio alfacsensis*	6	14.75	1.44	6	13.25	274.00
chemoheterotrophy	6	10.92	0.00	6	11.03	0.00	*Salmonella enterica*	19	22.00	60.93	7	12.25	0.00
Vibrio harveyi	5	10.67	36.00	2	8.03	0.00	*Pseudomonas aeruginosa*	18	21.50	50.99	7	12.25	0.00
Synechococcus sp. WH 7803	4	9.92	0.00	4	10.03	0.00	*Pseudomonas fluorescens*	17	21.33	32.20	7	12.25	0.00
Vibrio cyclitrophicus	3	9.92	68.00	2	2.83	0.00	*Pseudomonas chlororaphis*	15	20.33	19.06	7	12.25	0.00
Vibrio alginolyticus	3	9.67	0.00	3	10.50	5.00	*Pseudomonas putida*	14	19.50	3.71	7	12.25	0.00
Pseudoalteromonas marina	2	7.28	0.00	3	3.50	2.00	*Pseudomonas monteilii*	14	19.50	5.75	7	12.25	0.00
Pseudoalteromonas sp. 13-15	2	7.28	0.00	3	3.50	2.00	*Stenotrophomonas acidaminiphila*	13	19.17	10.11	7	12.25	0.00
Pseudoalteromonas spongiae	1	6.98	0.00	/	/	/	*Variovorax* sp. PMC12	1	1.00	0.00	3	11.67	0.00
Candidatus Pelagibacter sp. FZCC0015	4	4.00	10.00	4	9.25	18.00	*Vibrio owensii*	5	14.08	0.50	5	11.05	8.00

Candidatus Pelagibacter sp. HIMB1321	2	3.00	0.00	5	11.67	128.00	fermentation	/	/	/	5	10.88	50.00
alpha proteobacterium HIMB59	2	3.00	0.00	3	8.75	0.00	*Vibrio rotiferianus*	12	18.08	18.26	4	10.38	0.00
Paenibacillus larvae	1	2.50	0.00	4	9.25	18.00	*Vibrio harveyi*	4	13.58	0.00	4	10.38	0.00
Candidatus Pelagibacter ubique	1	2.50	0.00	2	6.92	0.00	*Pseudomonas* sp. LTGT-11-2Z	14	19.67	15.47	3	9.88	50.00
Marivivens sp. JLT3646	1	1.00	0.00	3	3.50	6.00	phototrophy	17	21.33	75.67	3	9.83	0.00
Rhodobacteracea e bacterium	1	1.00	0.00	1	2.33	0.00	photoautotroph y	15	20.33	19.18	3	9.83	0.00
							JJMV F	7	16.00	7.09	1	7.52	0.00
							Paenibacillus larvae	1	1.00	0.00	1	7.18	0.00
							aerobic_chemoh eterotrophy	13	19.00	28.49	1	1.00	0.00
							chemoheterotro phy	10	17.50	18.23	1	1.00	0.00

Table S3. Correlation between microbial species and function in the seawater of mariculture areas.

source	target	correlation	p-value	correlation_type
Vibrio sp. THAF190c	photosynthetic_cyanobacteria (Function)	-0.71023442	1.09E-08	negative
	oxygenic_photoautotrophy (Function)	-0.71023442	1.09E-08	negative
	photoautotrophy (Function)	-0.711987803	9.79E-09	negative
	phototrophy (Function)	-0.712978845	9.21E-09	negative
	fermentation (Function)	0.851419859	4.81E-15	positive
	nitrate_reduction (Function)	0.847074519	9.32E-15	positive
	animal_parasites_or_symbionts (Function)	0.749723652	6.36E-10	positive
Vibrio mediterranei	*Vibrio* sp. THAF190c	0.87352773	1.16E-16	positive
	fermentation (Function)	0.817038308	5.93E-13	positive
	Vibrio chagasii	0.797293692	6.05E-12	positive
	nitrate_reduction (Function)	0.793177054	9.42E-12	positive
	Vibrio harveyi	0.734171908	2.03E-09	positive
	animal_parasites_or_symbionts (Function)	0.707185058	1.33E-08	positive
Vibrio harveyi	*Vibrio* sp. THAF190c	0.728759291	2.93E-09	positive
	nitrate_reduction (Function)	0.708633505	1.21E-08	positive
	animal_parasites_or_symbionts (Function)	0.704364399	1.62E-08	positive
Vibrio chagasii	fermentation (Function)	0.89731275	7.52E-19	positive
	nitrate_reduction (Function)	0.870097198	2.06E-16	positive

	Vibrio sp. THAF190c	0.857594816	1.78E-15	positive
	Vibrio cyclitrophicus	0.787703215	1.64E-11	positive
	photoautotrophy (Function)	-0.757423289	3.20E-10	negative
	photosynthetic_cyanobacteria (Function)	-0.758261864	3.02E-10	negative
	oxygenic_photoautotrophy (Function)	-0.758261864	3.02E-10	negative
	phototrophy (Function)	-0.7621498	2.19E-10	negative
	aerobic_chemoheterotrophy (Function)	0.700781399	2.08E-08	positive
	nitrate_reduction (Function)	0.859805603	1.26E-15	positive
Vibrio alginolyticus	fermentation (Function)	0.816123499	6.48E-13	positive
	animal_parasites_or_symbionts (Function)	0.724261483	3.97E-09	positive
Synechococcus sp. WH 8101	photosynthetic_cyanobacteria (Function)	0.978654469	9.06E-36	positive
	oxygenic_photoautotrophy (Function)	0.978654469	9.06E-36	positive
	phototrophy (Function)	0.978273299	1.30E-35	positive
	photoautotrophy (Function)	0.978120831	1.42E-35	positive
	fermentation (Function)	-0.72723461	3.25E-09	negative
	Vibrio chagasii	-0.734705546	1.98E-09	negative
	aerobic_chemoheterotrophy (Function)	-0.915608919	6.01E-21	negative
	chemoheterotrophy (Function)	-0.921936345	8.97E-22	negative
Synechococcus	photoautotrophy (Function)	0.731961121	2.40E-09	positive

sp. WH 7803	photosynthetic_cyanobacteria (Function)	0.731275014	2.46E-09	positive
	oxygenic_photoautotrophy (Function)	0.731275014	2.46E-09	positive
	phototrophy (Function)	0.722813036	4.39E-09	positive
Pseudoalteromonas spongiae	*Vibrio harveyi*	0.721821994	4.68E-09	positive
Pseudoalteromonas sp. 13-15	*Pseudoalteromonas marina*	0.996721936	1.12E-56	positive
	Vibrio cyclitrophicus	0.72488517	3.83E-09	positive
Pseudoalteromonas marina	*Vibrio cyclitrophicus*	0.727096	3.25E-09	positive
phototrophy (Function)	photoautotrophy (Function)	0.999085192	7.85E-71	positive
	photosynthetic_cyanobacteria (Function)	0.999008957	4.19E-70	positive
	oxygenic_photoautotrophy (Function)	0.999008957	4.19E-70	positive
	nitrate_reduction (Function)	-0.737297503	1.62E-09	negative
	fermentation (Function)	-0.742100248	1.15E-09	negative
photosynthetic_cyanobacteria (Function)	oxygenic_photoautotrophy (Function)	1	0	positive
	nitrate_reduction (Function)	-0.73912712	1.41E-09	negative
	fermentation (Function)	-0.74271012	1.11E-09	negative
photoautotrophy (Function)	photosynthetic_cyanobacteria (Function)	0.999466362	8.62E-77	positive
	oxygenic_photoautotrophy (Function)	0.999466362	8.62E-77	positive

	nitrate_reduction (Function)	-0.739660759	1.40E-09	negative
	fermentation (Function)	-0.742786354	1.11E-09	negative
oxygenic_photoautotrophy (Function)	nitrate_reduction (Function)	-0.73912712	1.41E-09	negative
	fermentation (Function)	-0.74271012	1.11E-09	negative
nitrate_reduction (Function)	animal_parasites_or_symbionts (Function)	0.838383838	3.30E-14	positive
Marivivens sp. JLT3646	Rhodobacteraceae bacterium	0.846845817	9.36E-15	positive
fermentation (Function)	nitrate_reduction (Function)	0.955708024	9.20E-28	positive
	animal_parasites_or_symbionts (Function)	0.872307986	1.41E-16	positive
chemoheterotrophy (Function)	aerobic_chemoheterotrophy (Function)	0.993367639	8.56E-49	positive
	photosynthetic_cyanobacteria (Function)	-0.934286259	1.32E-23	negative
	oxygenic_photoautotrophy (Function)	-0.934286259	1.32E-23	negative
	photoautotrophy (Function)	-0.935658472	9.59E-24	negative
	phototrophy (Function)	-0.936878216	6.32E-24	negative
Candidatus Pelagibacter ubique	*Candidatus Pelagibacter* sp. FZCC0015	0.88580141	9.88E-18	positive
Candidatus Pelagibacter	*alpha proteobacterium* HIMB59	0.822603392	2.94E-13	positive
	Candidatus Pelagibacter sp.	0.78951782	1.38E-11	positive

sp. HIMB1321	FZCC0015			
Candidatus Pelagibacter sp. FZCC0015	*alpha proteobacterium* HIMB59	0.797751096	5.89E-12	positive
	Paenibacillus larvae	0.729216695	2.87E-09	positive
aerobic_chemoheterotrophy (Function)	photosynthetic_cyanobacteria (Function)	-0.934133791	1.32E-23	negative
	oxygenic_photoautotrophy (Function)	-0.934133791	1.32E-23	negative
	photoautotrophy (Function)	-0.935506003	9.59E-24	negative
	phototrophy (Function)	-0.937030684	6.32E-24	negative

Table S4. Correlation between microbial species and function in the seawater of non-mariculture areas.

source	target	correlation	p-value	correlation_type
Vibrio sp. THAF190c	fermentation (Function)	0.806289308	2.09E-12	positive
	nitrate_reduction (Function)	0.773203735	6.47E-11	positive
Vibrio mediterranei	*Vibrio chagasii*	0.7117591	9.43E-09	positive
	Tenacibaculum mesophilum	0.701772441	1.91E-08	positive
	Vibrio harveyi (Function)	0.750104822	5.45E-10	positive
	Vibrio sp. THAF190c	0.886716219	7.51E-18	positive
Vibrio harveyi	*Vibrio* sp. THAF190c	0.750943396	5.15E-10	positive
	Tenacibaculum mesophilum	0.747360396	6.37E-10	positive
Vibrio chagasii	*Vibrio* sp. THAF190c	0.821002478	3.44E-13	positive
	phototrophy (Function)	-0.718696398	5.99E-09	negative
	photoautotrophy (Function)	-0.71930627	5.80E-09	negative
	photosynthetic_cyanobacteria (Function)	-0.717705355	6.29E-09	negative
	oxygenic_photoautotrophy (Function)	-0.717705355	6.29E-09	negative
	fermentation (Function)	0.916447494	4.49E-21	positive
	nitrate_reduction (Function)	0.895940537	9.64E-19	positive
Vibrio alginolyticus	fermentation (Function)	0.714274824	7.88E-09	positive
	nitrate_reduction (Function)	0.788831713	1.33E-11	positive
Tenacibaculum mesophilum	*Vibrio* sp. THAF190c	0.724871355	3.90E-09	positive
	phototrophy (Function)	-0.710005718	1.06E-08	negative

	photoautotrophy (Function)	-0.719839909	5.64E-09	negative
	photosynthetic_cyanobacteria (Function)	-0.727920717	3.09E-09	negative
	oxygenic_photoautotrophy (Function)	-0.727920717	3.09E-09	negative
	fermentation (Function)	0.732875929	2.15E-09	positive
	nitrate_reduction (Function)	0.709548313	1.09E-08	positive
	Tenacibaculum mesophilum	-0.735010482	1.83E-09	negative
	chemoheterotrophy (Function)	-0.923765962	5.73E-22	negative
	aerobic_chemoheterotrophy (Function)	-0.925900515	2.97E-22	negative
Synechococcus sp. WH 8101	phototrophy (Function)	0.971945874	8.48E-33	positive
	photoautotrophy (Function)	0.972479512	5.65E-33	positive
	photosynthetic_cyanobacteria (Function)	0.972936916	4.04E-33	positive
	oxygenic_photoautotrophy (Function)	0.972936916	4.04E-33	positive
	fermentation (Function)	-0.763445779	1.66E-10	negative
	nitrate_reduction (Function)	-0.715494568	7.36E-09	negative
Synechococcus sp. WH 7803	phototrophy (Function)	0.749266247	5.77E-10	positive
	photoautotrophy (Function)	0.748885077	5.86E-10	positive
	photosynthetic_cyanobacteria (Function)	0.74743663	6.37E-10	positive

	oxygenic_photoautotrophy (Function)	0.74743663	6.37E-10	positive
Pseudoalteromonas sp. 13-15	*Pseudoalteromonas marina*	0.990165809	2.31E-44	positive
	Vibrio cyclitrophicus	0.804383457	2.57E-12	positive
Pseudoalteromonas marina	*Vibrio cyclitrophicus*	0.79858967	4.70E-12	positive
phototrophy (Function)	photoautotrophy (Function)	0.999008957	6.28E-70	positive
	photosynthetic_cyanobacteria (Function)	0.997865447	1.90E-61	positive
	oxygenic_photoautotrophy (Function)	0.997865447	1.90E-61	positive
	fermentation (Function)	-0.830379264	1.04E-13	negative
	nitrate_reduction (Function)	-0.797141224	5.34E-12	negative
photosynthetic_cyanobacteria (Function)	oxygenic_photoautotrophy (Function)	1	0	positive
	fermentation (Function)	-0.827253669	1.52E-13	negative
	nitrate_reduction (Function)	-0.798437202	4.70E-12	negative
photoautotrophy (Function)	photosynthetic_cyanobacteria (Function)	0.999161426	1.09E-71	positive
	oxygenic_photoautotrophy (Function)	0.999161426	1.09E-71	positive
	fermentation (Function)	-0.830379264	1.04E-13	negative
	nitrate_reduction (Function)	-0.798665904	4.70E-12	negative

Paenibacillus larvae	*alpha proteobacterium* HIMB59	0.815437393	6.89E-13	positive
oxygenic_photoautotrophy (Function)	fermentation (Function)	-0.827253669	1.52E-13	negative
	nitrate_reduction (Function)	-0.798437202	4.70E-12	negative
Marivivens sp. JLT3646	*Rhodobacteraceae bacterium*	0.846312178	1.05E-14	positive
	Pseudoalteromonas sp. 13-15	0.781284544	2.87E-11	positive
	Pseudoalteromonas marina	0.788450543	1.35E-11	positive
fermentation (Function)	nitrate_reduction (Function)	0.955098151	1.30E-27	positive
chemoheterotrophy (Function)	aerobic_chemoheterotrophy (Function)	0.993291405	1.32E-48	positive
	phototrophy (Function)	-0.921326472	1.20E-21	negative
	photoautotrophy (Function)	-0.920411664	1.52E-21	negative
	photosynthetic_cyanobacteria (Function)	-0.919420621	1.90E-21	negative
	oxygenic_photoautotrophy (Function)	-0.919420621	1.90E-21	negative
Candidatus Pelagibacter ubique	*Candidatus Pelagibacter* sp. FZCC0015	0.905469792	9.30E-20	positive
	Paenibacillus larvae	0.728378121	3.07E-09	positive
	Vibrio alginolyticus	-0.7153421	7.36E-09	negative
Candidatus Pelagibacter sp. HIMB1321	*Candidatus Pelagibacter* sp. FZCC0015	0.856756242	1.99E-15	positive
	Paenibacillus larvae	0.760396417	2.18E-10	positive
	alpha proteobacterium	0.875814751	6.64E-17	positive

	HIMB59			
	nitrate_reduction (Function)	-0.721898228	4.87E-09	negative
Candidatus Pelagibacter sp. FZCC0015	*Paenibacillus larvae*	0.857747284	1.74E-15	positive
	alpha proteobacterium HIMB59	0.876424624	6.09E-17	positive
aerobic_chemoheterotrophy (Function)	phototrophy (Function)	-0.934057557	2.04E-23	negative
	photoautotrophy (Function)	-0.932075472	4.01E-23	negative
	photosynthetic_cyanobacteria (Function)	-0.930245855	6.90E-23	negative
	oxygenic_photoautotrophy (Function)	-0.930245855	6.90E-23	negative

Table S5. Correlation between microbial species and function in the sediment of mariculture areas

source	target	correlation	p-value	correlation_type
Stenotrophomonas acidaminiphila	*Pseudomonas* sp. LTGT-11-2Z	0.92862424	4.33E-06	positive
	Pseudomonas aeruginosa	0.799117214	0.000945306	positive
	Pseudomonas putida	0.927152569	4.75E-06	positive
	Agrobacterium tumefaciens	0.856512373	0.000161911	positive
	Pseudomonas fluorescens	0.92862424	4.33E-06	positive
	Pseudomonas chlororaphis	0.946284289	9.32E-07	positive
	Mesorhizobium soli	0.75055208	0.003226482	positive
	Pseudomonas monteilii	0.924889543	5.37E-06	positive
	Salmonella enterica	0.812362251	0.000643159	positive
	phototrophy (Function)	-0.880059105	6.56E-05	negative
	photoautotrophy (Function)	-0.943340947	1.25E-06	negative
	photosynthetic_cyanobacteria (Function)	-0.913907532	1.12E-05	negative
	oxygenic_photoautotrophy (Function)	-0.913907532	1.12E-05	negative
Pseudomonas sp. LTGT-11-2Z	*Pseudomonas aeruginosa*	0.741176471	0.003722977	positive
	Pseudomonas putida	0.820588235	0.000535119	positive
	Agrobacterium tumefaciens	0.741176471	0.003722977	positive
	Pseudomonas fluorescens	0.858823529	0.000150882	positive
	Pseudomonas chlororaphis	0.852941176	0.000182093	positive
	Mesorhizobium soli	0.720588235	0.005439795	positive
	Pseudomonas monteilii	0.828550629	0.000436488	positive
	Salmonella enterica	0.744117647	0.003618447	positive

	Vibrio rotiferianus	0.714705882	0.006046958	positive
	phototrophy (Function)	-0.855882353	0.000161911	negative
	photoautotrophy (Function)	-0.902941176	2.09E-05	negative
	photosynthetic_cyanobacteria (Function)	-0.8	0.0009288	negative
	oxygenic_photoautotrophy (Function)	-0.8	0.0009288	negative
Pseudomonas aeruginosa	*Pseudomonas putida*	0.882352941	5.89E-05	positive
	Vibrio harveyi	0.826470588	0.000459566	positive
	Agrobacterium tumefaciens	0.861764706	0.000133539	positive
	Pseudomonas fluorescens	0.905882353	1.74E-05	positive
	Pseudomonas chlororaphis	0.864705882	0.000117849	positive
	Vibrio owensii	0.732352941	0.004264897	positive
	Vibrio alfacsensis	0.720588235	0.005439795	positive
	Pseudomonas monteilii	0.819720605	0.000545326	positive
	Salmonella enterica	0.970588235	2.28E-08	positive
	Vibrio rotiferianus	0.876470588	7.65E-05	positive
	chemoheterotrophy (Function)	0.741176471	0.003722977	positive
	aerobic_chemoheterotrophy (Function)	0.782352941	0.001514602	positive
	phototrophy (Function)	-0.764705882	0.002340954	negative
	photoautotrophy (Function)	-0.773529412	0.001873723	negative
	photosynthetic_cyanobacteria (Function)	-0.702941176	0.007416603	negative
	oxygenic_photoautotrophy (Function)	-0.702941176	0.007416603	negative

	Agrobacterium tumefaciens	0.973529412	1.41E-08	positive
	Pseudomonas fluorescens	0.973529412	1.41E-08	positive
	Pseudomonas chlororaphis	0.985294118	5.56E-10	positive
	Pseudomonas monteilii	0.940397606	1.66E-06	positive
	Salmonella enterica	0.882352941	5.89E-05	positive
	Vibrio rotiferianus	0.732352941	0.004264897	positive
Pseudomonas putida	aerobic_chemoheterotrophy (Function)	0.735294118	0.004110103	positive
	phototrophy (Function)	-0.885294118	5.21E-05	negative
	photoautotrophy (Function)	-0.905882353	1.74E-05	negative
	photosynthetic_cyanobacteria (Function)	-0.867647059	0.000107531	negative
	oxygenic_photoautotrophy (Function)	-0.867647059	0.000107531	negative
	Vibrio owensii	0.817647059	0.000553174	positive
Vibrio harveyi	*Salmonella enterica*	0.802941176	0.00086291	positive
	Vibrio rotiferianus	0.811764706	0.000649225	positive
	photosynthetic_cyanobacteria (Function)	0.817647059	0.000553174	positive
Synechococcus sp. WH 8101	oxygenic_photoautotrophy (Function)	0.817647059	0.000553174	positive
	animal_parasites_or_symbionts (Function)	-0.720588235	0.005439795	negative
	nitrogen_respiration (Function)	-0.735294118	0.004110103	negative
Agrobacterium tumefaciens	*Pseudomonas fluorescens*	0.929411765	4.33E-06	positive
	Pseudomonas chlororaphis	0.947058824	9.09E-07	positive

	Pseudomonas monteilii	0.8903608	4.24E-05	positive
	Salmonella enterica	0.838235294	0.000315869	positive
	Vibrio rotiferianus	0.752941176	0.003065465	positive
	phototrophy (Function)	-0.835294118	0.000340577	negative
	photoautotrophy (Function)	-0.85	0.000201172	negative
	photosynthetic_cyanobacteria (Function)	-0.823529412	0.000486712	negative
	oxygenic_photoautotrophy (Function)	-0.823529412	0.000486712	negative
Pseudomonas fluorescens	*Pseudomonas chlororaphis*	0.982352941	1.19E-09	positive
	Mesorhizobium soli	0.717647059	0.005715622	positive
	Pseudomonas monteilii	0.962472667	1.01E-07	positive
	Salmonella enterica	0.920588235	6.96E-06	positive
	Vibrio rotiferianus	0.782352941	0.001514602	positive
	chemoheterotrophy (Function)	0.779411765	0.00163314	positive
	aerobic_chemoheterotrophy (Function)	0.826470588	0.000459566	positive
	phototrophy (Function)	-0.920588235	6.96E-06	negative
	JJMV F (Function)	0.741176471	0.003722977	positive
	photoautotrophy (Function)	-0.923529412	5.82E-06	negative
	photosynthetic_cyanobacteria (Function)	-0.867647059	0.000107531	negative
	oxygenic_photoautotrophy (Function)	-0.867647059	0.000107531	negative
Pseudomonas chlororaphis	*Mesorhizobium soli*	0.717647059	0.005715622	positive
	Pseudomonas monteilii	0.96983102	2.45E-08	positive

	Salmonella enterica	0.867647059	0.000107531	positive
	Vibrio rotiferianus	0.711764706	0.006393028	positive
	aerobic_chemoheterotrophy (Function)	0.744117647	0.003618447	positive
	phototrophy (Function)	-0.894117647	3.54E-05	negative
	photoautotrophy (Function)	-0.905882353	1.74E-05	negative
	photosynthetic_cyanobacteria (Function)	-0.864705882	0.000117849	negative
	oxygenic_photoautotrophy (Function)	-0.864705882	0.000117849	negative
Vibrio owensii	*Vibrio alfacsensis*	0.891176471	4.14E-05	positive
	Salmonella enterica	0.708823529	0.006656387	positive
	Vibrio rotiferianus	0.817647059	0.000553174	positive
Mesorhizobium soli	*Bradyrhizobium* sp. 6(2017)	0.844117647	0.00025142	positive
	Bradyrhizobium sp. SK17	0.835294118	0.000340577	positive
	Mycolicibacterium aubagnense	0.732352941	0.004264897	positive
	phototrophy (Function)	-0.764705882	0.002340954	negative
	photoautotrophy (Function)	-0.773529412	0.001873723	negative
Vibrio alfacsensis	*Salmonella enterica*	0.75	0.003241794	positive
	Vibrio rotiferianus	0.773529412	0.001873723	positive
	chemoheterotrophy (Function)	0.732352941	0.004264897	positive
	aerobic_chemoheterotrophy (Function)	0.755882353	0.002896815	positive
Pseudomonas monteilii	*Salmonella enterica*	0.824135617	0.000486712	positive
	aerobic_chemoheterotrophy (Function)	0.744665397	0.003618447	positive

	phototrophy (Function)	-0.878587434	6.96E-05	negative
	JJMV F (Function)	0.700515274	0.007739352	positive
	photoautotrophy (Function)	-0.887417459	4.70E-05	negative
	photosynthetic_cyanobacteria (Function)	-0.887417459	4.70E-05	negative
	oxygenic_photoautotrophy (Function)	-0.887417459	4.70E-05	negative
Salmonella enterica	*Vibrio rotiferianus*	0.844117647	0.00025142	positive
	chemoheterotrophy (Function)	0.814705882	0.000599655	positive
	aerobic_chemoheterotrophy (Function)	0.85	0.000201172	positive
	phototrophy (Function)	-0.835294118	0.000340577	negative
	JJMV F (Function)	0.741176471	0.003722977	positive
	photoautotrophy (Function)	-0.823529412	0.000486712	negative
	photosynthetic_cyanobacteria (Function)	-0.735294118	0.004110103	negative
	oxygenic_photoautotrophy (Function)	-0.735294118	0.004110103	negative
Paenibacillus larvae	*Variovorax* sp. PMC12	0.926470588	4.84E-06	positive
Bradyrhizobium sp. 6(2017)	*Bradyrhizobium* sp. SK17	0.991176471	2.37E-11	positive
	Mycolicibacterium aubagnense	0.905882353	1.74E-05	positive
Vibrio rotiferianus	chemoheterotrophy (Function)	0.744117647	0.003618447	positive
	aerobic_chemoheterotrophy (Function)	0.782352941	0.001514602	positive
Bradyrhizobiu	*Mycolicibacterium aubagnense*	0.897058824	3.01E-05	positive

m sp. SK17	phototrophy (Function)	-0.708823529	0.006656387	negative
chemoheterotrophy (Function)	aerobic_chemoheterotrophy (Function)	0.982352941	1.19E-09	positive
	phototrophy (Function)	-0.788235294	0.001311711	negative
	JJMV F (Function)	0.908823529	1.60E-05	positive
	photoautotrophy (Function)	-0.708823529	0.006656387	negative
	animal_parasites_or_symbionts (Function)	0.758823529	0.002709554	positive
aerobic_chemoheterotrophy (Function)	phototrophy (Function)	-0.817647059	0.000553174	negative
	JJMV F (Function)	0.947058824	9.09E-07	positive
	photoautotrophy (Function)	-0.752941176	0.003065465	negative
	animal_parasites_or_symbionts (Function)	0.814705882	0.000599655	positive
phototrophy (Function)	JJMV F (Function)	-0.735294118	0.004110103	negative
	photoautotrophy (Function)	0.970588235	2.28E-08	positive
	photosynthetic_cyanobacteria (Function)	0.855882353	0.000161911	positive
	oxygenic_photoautotrophy (Function)	0.855882353	0.000161911	positive
JJMV F (Function)	animal_parasites_or_symbionts (Function)	0.873529412	8.76E-05	positive
photoautotrophy (Function)	photosynthetic_cyanobacteria (Function)	0.932352941	3.51E-06	positive
	oxygenic_photoautotrophy (Function)	0.932352941	3.51E-06	positive
photosynthe	oxygenic_photoautotrophy	1	0	positive

tic_cyanobacteria (Function)	(Function)			

Table S6. Correlation between microbial species and function in the sediment of non-mariculture area

source	target	correlation	p-value	correlation_type
Mesorhizobium soli	*Bradyrhizobium* sp. 6(2017)	0.824561404	0.000253172	positive
	Mycolicibacterium aubagnense	0.752321981	0.001963768	positive
	Bradyrhizobium sp. SK17	0.82249742	0.000264084	positive
	Variovorax sp. PMC12	0.795665635	0.000658094	positive
	Agrobacterium tumefaciens	0.706914345	0.005781918	positive
	nitrogen_fixation (Function)	0.789473684	0.00078792	positive
	ureolysis (Function)	0.950464396	8.48E-08	positive
	photosynthetic_cyanobacteria (Function)	-0.758513932	0.001711732	negative
	oxygenic_photoautotrophy (Function)	-0.758513932	0.001711732	negative
Paenibacillus larvae	*Pseudomonas* sp. LTGT-11-2Z	0.706914345	0.005781918	positive
Bradyrhizobium sp. 6(2017)	*Mycolicibacterium aubagnense*	0.880288958	2.10E-05	positive
	Bradyrhizobium sp. SK17	0.997936017	3.58E-18	positive
	nitrogen_fixation (Function)	0.902992776	7.41E-06	positive
	ureolysis (Function)	0.849329205	9.95E-05	positive
	photosynthetic_cyanobacteria (Function)	-0.750257998	0.002025759	negative

	oxygenic_photoautotrophy (Function)	-0.750257998	0.002025759	negative
Mycolicibacterium aubagnense	*Bradyrhizobium* sp. SK17	0.892672859	1.09E-05	positive
	nitrogen_fixation (Function)	0.824561404	0.000253172	positive
	ureolysis (Function)	0.752321981	0.001963768	positive
	photosynthetic_cyanobacteria (Function)	-0.766769866	0.001464411	negative
	oxygenic_photoautotrophy (Function)	-0.766769866	0.001464411	negative
Bradyrhizobium sp. SK17	nitrogen_fixation (Function)	0.900928793	7.80E-06	positive
	ureolysis (Function)	0.845201238	0.000115377	positive
	photosynthetic_cyanobacteria (Function)	-0.758513932	0.001711732	negative
	oxygenic_photoautotrophy (Function)	-0.758513932	0.001711732	negative
Variovorax sp. PMC12	nitrogen_fixation (Function)	0.700722394	0.006602005	positive
	ureolysis (Function)	0.787409701	0.000830537	positive
Salmonella enterica	*Pseudomonas aeruginosa*	0.805985552	0.000468202	positive
	Pseudomonas putida	0.878224974	2.24E-05	positive
	Pseudomonas fluorescens	0.839009288	0.000143366	positive
	Pseudomonas chlororaphis	0.888544892	1.35E-05	positive
	Agrobacterium tumefaciens	0.892672859	1.09E-05	positive
	Pseudomonas monteilii	0.752321981	0.001963768	positive
	Stenotrophomonas acidaminiphila	0.818369453	0.000299997	positive
Pseudomon	*Pseudomonas putida*	0.878224974	2.24E-05	positive

as	*Pseudomonas fluorescens*	0.888544892	1.35E-05	positive
aeruginosa	*Pseudomonas chlororaphis*	0.900928793	7.80E-06	positive
	Agrobacterium tumefaciens	0.7750258	0.001187534	positive
	Pseudomonas monteilii	0.832817337	0.000185413	positive
	Stenotrophomonas acidaminiphila	0.861713106	5.52E-05	positive
	Vibrio harveyi	0.95872033	2.31E-08	positive
Vibrio	*Vibrio owensii*	0.785345717	0.000875083	positive
rotiferianus	*Vibrio alfacsensis*	0.77542602	0.001187534	positive
	fermentation (Function)	0.729618163	0.003462671	positive
Vibrio	*Vibrio owensii*	0.847265222	0.000107162	positive
harveyi	*Vibrio alfacsensis*	0.816727006	0.000314081	positive
	fermentation (Function)	0.770897833	0.001331224	positive
	Pseudomonas fluorescens	0.962848297	1.41E-08	positive
	Pseudomonas chlororaphis	0.981424149	9.75E-11	positive
Pseudomon	*Agrobacterium tumefaciens*	0.839009288	0.000143366	positive
as putida	*Pseudomonas monteilii*	0.90505676	6.99E-06	positive
	Stenotrophomonas acidaminiphila	0.931888545	6.81E-07	positive
	Pseudomonas chlororaphis	0.977296182	3.60E-10	positive
Pseudomon	*Agrobacterium tumefaciens*	0.789473684	0.00078792	positive
as	*Pseudomonas monteilii*	0.919504644	2.13E-06	positive
fluorescens	*Stenotrophomonas acidaminiphila*	0.894736842	1.03E-05	positive
Pseudomon	*Agrobacterium tumefaciens*	0.841073271	0.000136873	positive
as	*Pseudomonas monteilii*	0.896800826	9.21E-06	positive

chlororaphis	*Stenotrophomonas acidaminiphila*	0.925696594	1.24E-06	positive
Agrobacterium tumefaciens	*Pseudomonas monteilii*	0.82249742	0.000264084	positive
	Stenotrophomonas acidaminiphila	0.874097007	2.80E-05	positive
Vibrio owensii	*Vibrio alfacsensis*	0.904491602	6.99E-06	positive
	Pseudomonas sp. LTGT-11-2Z	0.708978328	0.005648852	positive
	fermentation (Function)	0.725490196	0.003801519	positive
Pseudomonas monteilii	*Stenotrophomonas acidaminiphila*	0.884416925	1.72E-05	positive
Vibrio alfacsensis	*Pseudomonas* sp. LTGT-11-2Z	0.805369235	0.000469838	positive
	fermentation (Function)	0.81982458	0.000288692	positive
	nitrogen_fixation (Function)	-0.746515329	0.00222151	negative
chemoheterotrophy (Function)	aerobic_chemoheterotrophy (Function)	0.880288958	2.10E-05	positive
fermentation (Function)	JJMV F (Function)	0.933952528	5.85E-07	positive
nitrogen_fixation (Function)	ureolysis (Function)	0.849329205	9.95E-05	positive
ureolysis (Function)	photosynthetic_cyanobacteria (Function)	-0.764705882	0.001510714	negative
	oxygenic_photoautotrophy (Function)	-0.764705882	0.001510714	negative

	photoautotrophy (Function)	0.95872033	2.31E-08	positive
phototrophy (Function)	photosynthetic_cyanobacteria (Function)	0.896800826	9.21E-06	positive
	oxygenic_photoautotrophy (Function)	0.896800826	9.21E-06	positive
photoautotrophy (Function)	photosynthetic_cyanobacteria (Function)	0.933952528	5.85E-07	positive
	oxygenic_photoautotrophy (Function)	0.933952528	5.85E-07	positive
photosynthetic_cyanobacteria (Function)	oxygenic_photoautotrophy (Function)	1	0	positive
